# Comparative Assessment of the Utility of Anthropometric and Bioelectrical Impedance Indices as Potential Predictors of Hypertension within a Ghanaian Adult Population: A Cross-Sectional Study

**DOI:** 10.1155/2022/2242901

**Published:** 2022-01-27

**Authors:** Adjoa Agyemang Boakye, David Adedia, Gaston Kofi Hunkpe, Rosina Afua Ampomah Carr, Veronica Fafali Ami AdanusahAll, Bless Seyram Agbenyo, Kwabena Obeng Duedu

**Affiliations:** ^1^Department of Biomedical Sciences, School of Basic & Biomedical Sciences, University of Health & Allied Sciences, Ho, Ghana; ^2^Department of Basic Sciences, School of Basic & Biomedical Sciences, University of Health & Allied Sciences, Ho, Ghana

## Abstract

**Background:**

Hypertension is an important public health concern that is claiming millions of lives worldwide. In sub-Saharan African countries, where some of the highest prevalence rates are being recorded, sufficient attention has not been given to its control.

**Objective:**

The aim of this study was to determine the association and predictive potential of different anthropometric and bioelectrical impedance analysis (BIA) measures for hypertension.

**Methods:**

A total of 812 individuals (204 men and 608 women) were enrolled, and their blood pressure measurement was determined. Direct anthropometric measures (weight, height, waist circumference (WC), and hip circumference) and derived anthropometric measures (body mass index, conicity index, abdominal volume index (AVI), and body adiposity index) were determined. BIA indices investigated included visceral fat level (VF), percentage body fat (%BF), resting metabolic rate (RMR), and skeletal muscle mass.

**Results:**

A prevalence of 31.28% was observed for hypertension in the total study population, with males having a slightly higher prevalence than females. Except for the skeletal muscle mass, all the other indices measured showed an increasing trend from normotension to prehypertension and hypertension. Age and visceral fat level showed the highest correlation with systolic blood pressure for both genders. Receiver operator characteristic analysis showed that age was the best predictor of hypertension in both genders, whereas, in predicting prehypertension, RMR was the best predictor in males, and WC was the best predictor in females. VF, WC, and AVI were other good predictors of hypertension in this study population. However, BMI and % BF had a low predictive value for hypertension.

**Conclusion:**

The result of this study shows that within this study population in addition to age, measures of central obesity rather than general obesity are the likely drivers of the hypertension epidemic; thus, measures aimed at controlling central obesity may offer some therapeutic and preventive advantage.

## 1. Introduction 

Hypertension, diagnosed as a systolic blood pressure of ≥140 mmHg and/or diastolic blood pressure of ≥90 mmHg, is a significant risk factor for the development of stroke, heart failure, peripheral vascular disease, and chronic kidney diseases [1]. The World Health Organization estimates that approximately 1.13 billion people worldwide are living with hypertension and that only 20% of these individuals have their blood pressures under control, making hypertension the single largest contributor to the global burden of disease [2, 3]. Currently, some of the highest prevalence rates for hypertension are being recorded in sub-Saharan Africa, a region that is already struggling with infectious diseases, such as malaria, and lack of healthcare facilities and personnel [4–6]. It has resulted in calls by the African Union and United Nations to make hypertension a high priority research area, however, public health response from many African nations remains inadequate, leading to low awareness and poor disease management [7–9].

Hypertension is a multifactorial disease, with aging, sedentary lifestyle, smoking, and high caloric diet intake being important risk factors [10]. Mechanistic studies show that oxidative stress, changes in circulating immune cell populations, dyslipidemia, imbalances in serum electrolyte content, and genetics are key players in the development and progression of hypertension [11–13]. Thus, t management of hypertension involves a combination of medication and lifestyle changes. However, about a third of persons living with hypertension fail to achieve proper blood pressure controls even when prescribed with three or more antihypertensives [14]. This observation, coupled with the high mortality and morbidity associated with hypertension, suggests that all risk factors be defined and their mechanism of action elucidated. Because of the limited medical infrastructure in most sub-Saharan African countries, identifying the risk factors that are easy to measure and still have a high-predictive/high-diagnostic potential is essential.

Several studies have reported an association between hypertension and anthropometric indices of obesity [15–18]. Recent advancements in technology have also resulted in the availability of potable bioelectric impedance analysis (BIA) tools that can provide information on the body composition of an individual, such as percentage body fat, visceral fat level, skeletal muscle mass, easily. The determination of association between hypertension, anthropometric indices, and BIA indices has been the subject of various pieces of research carried out across different racial and ethnic groups. However, the results from these studies have been inconclusive and controversial as far as identifying the best indices in predicting hypertension is concerned [19, 20]. For instance, in an Italian population-based study, the body mass index was seen as the single best predictor of the risk of hypertension [21]. A related study amongst Japanese showed that BMI was the best predictor of hypertension in women, whereas waist circumference was the best predictor in men [22]. The association of waist circumference and hypertension was, however, observed for both genders in a Greek study [23]. Amongst the Chinese, an association of blood pressure with waist circumference, and not BMI, has been reported even though other studies have shown an association between blood pressure and BMI [24–26].

These results from previous studies suggest that the predictive power of an anthropometric index may be population-dependent and may vary with age and gender. To develop tools aimed at preventing hypertension, the identification of population-specific risk factors may be important. The current study was designed to determine the prevalence of hypertension and the association between blood pressure and different anthropometric and BIA measures of adiposity amongst a Ghanaian population. It is to generate baseline data on these associations and possibly look into how to control these indices to curb this growing hypertension epidemic.

## 2. Materials and Methods

### 2.1. Study Design and Population

The study followed a cross-sectional design and was conducted in randomly selected communities within the Volta Region of Ghana. Announcements were made, and the purpose and nature of the study were explained to persons living within the catchment area. Consenting adults (persons ≥ 18 years) were enrolled in the study. Demographic, lifestyle, and socioeconomic status data were collected from participants using a well-structured questionnaire. Blood pressure measures, anthropometric impedance measurements, and bioelectrical impedance measurements were collected following standard procedures. The derived measures of adiposity, such as body mass index (BMI), body adiposity index (BAI), conicity index (CI), and abdominal volume index (AVI), were calculated from the anthropometric measures using appropriate formulas. The study was approved by the Research Ethics Committee of the University of Health and Allied Sciences.

### 2.2. Blood Pressure Measurement and Classification of Study Participants

Study participants were allowed to rest for at least 10 minutes before blood pressure measurements. Measurements were taken using a mercury sphygmomanometer, with the participant seated. The average of two measurements was used to estimate the BP. The diastolic values of ≥140 mmHg and/or systolic values of ≥90 mmHg were classified as hypertension. Systolic measurements from 120 to 139 mmHg and/or diastolic measurements from 80 to 89 were considered prehypertension, whereas systolic measurements <120 mmHg and diastolic measurements of <80 mmHg were considered normotensive.

### 2.3. Anthropometric and BIA Measurement

Height was measured with the respondents having no shoes on and not tipping heads up or down and corrected to the nearest 0.1 cm using a Stadiometer. Weight was measured to the nearest 0.1 kg using the Omron body composition analyzer (Omron Healthcare Co., Ltd., Kyoto, Japan). No adjustment for clothing was made, however, participants were encouraged to be lightly clothed for the weight determination. Age and gender were then input into the body composition analyzer prior to the determination of the visceral fat (VF), %body fat (BF), skeletal muscle mass (SM), and resting metabolic rate (RMR). These were obtained by following the manufacturer's instructions.

The derived measures of adiposity, such as CI, AVI, and BAI, were calculated using the subsequent formulas as previously described [27].

(1) Body Mass Index (BMI):
  (1) $$\begin{array}{c} \text{BMI}=\;\frac{\text{Weight }\left(\text{Kg}\right)}{\text{Height }{\left(\text{m}\right)}^{2}} \end{array}$$
(2) Body Adiposity Index (BAI):
  (2) $$\begin{array}{c} \text{BAI}=\frac{\text{HC}\left(\text{cm}\right)}{{\left[\text{Height}\left(\text{m}\right)\right]}^{1.5}}-18. \end{array}$$
(3) Abdominal Volume Index (AVI):
  (3) $$\begin{array}{c} \text{AVI}=\frac{\left[2{\left(\text{WC }\left(\text{cm}\right)\right)}^{2}+0.7{\left(\text{WC }\left(\text{cm}\right)-\text{HC}\left(\text{cm}\right)\right)}^{2}\right]}{1000}. \end{array}$$
(4) Conicity Index (CI):

(4) $$\begin{array}{c} \text{CI}=\frac{\text{WC}\left(\text{m}\right)}{\left[0.109\times \sqrt{\text{Weight }\left(\text{Kg}/\text{Height }\left(\text{m}\right)\right)}\right]}. \end{array}$$

### 2.4. Statistical Analysis

The data obtained from this study was analyzed using IMB Statistical Package for Social Scientist (SPSS) version 25 and XLSTAT. Descriptive statistics are given as mean ± standard deviation. Analysis of variance (ANOVA) and *t*-test were used when the parametric assumptions were satisfied whilst Kruskal–Wallis and Mann–Whitney tests were used for non-normal data. The Welch ANOVA and Games–Howell multiple comparisons were used for datasets that have heterogeneous variances. The strength of linear correlation between BP measures and the different anthropometric and BIA indices was carried out using the Pearson product-moment correlation. A *p*-value of 0.05 or below was considered statistically significant. To determine the predictive potentials of the various indices, the receiver operative characteristics (ROC) analysis was carried out. For these curves, sensitivity is plotted with false positive (1-specificity), where sensitivity refers to the probability of an anthropometric or bioelectrical impedance index classifying someone as having hypertension when the person is hypertensive. Specificity refers to the probability of an anthropometric or bioelectrical impedance index classifying someone as not having hypertension when the person is not hypertensive.

## 3. Results

A total of 812 individuals with a mean age of 47.39 years were included in this study. The overall prevalence of prehypertension and hypertension was 30.54% and 31.28%, respectively. The awareness level for hypertension was 33.07%. Of the study participants, 204 (25.12%) were males, whereas 608 (74.88%) were females. A prevalence of 33.82% was recorded in males for prehypertension and hypertension, respectively, whereas for females, the prevalence was 29.44% and 30.26% for prehypertension and hypertension, respectively. Of the data analyzed, 1.23%, 10.59%, 12.31%, 21.67%, and 54.19% were cohabiting, widowed, divorced, single, and married, respectively. In terms of their educational status, 15.39% had no formal education, 17.6% were primary school leavers, 38.18% had middle/Junior high school education, 16.26% were secondary/high school leavers, and 12.81% had tertiary education.

The general characteristics of the study population are presented in Tables 1 and 2. Generally, except for the skeletal muscle mass, the values of all the anthropometric and BIA measures showed an increasing trend from normotensive to prehypertensive and to hypertensive (Table 1). In Table 2, a similar trend was seen when the results were categorized according to gender. The indices that were higher in females than in males for all BP classes are as follows: waist circumference (WC), % body fat (%BF), body mass index (BMI), conicity index (CI), abdominal volume index (AVI), and body adiposity index (BAI), whereas resting metabolic rate (RMR) and skeletal muscle mass (SM) were higher in the males than in females for all BP classes. Age and visceral fat showed no trends when compared between the genders.

Table 3 shows the results of the correlation between BP and the anthropometric and BIA indices. All indices showed a significant correlation with the systolic BP (SBP) and diastolic BP (DBP) for the total population and both genders, except for resting metabolic rate and body adiposity index that showed no significant association in males. The highest correlation with SBP in all three groups was with age, followed by visceral fat. Correlation with DBP was quite heterogeneous with visceral fat, showing the highest correlation in the total and female populations, whiles the skeletal muscle mass showed the highest correlation in males.

The results of the predictive potential of each individual index in discriminating between the normotensive and hypertensive are presented in Table 4. The AUC values presented show that in both genders, age was the best predictor of hypertension, however, gender-based differences in the predictive power of the other indices were observed. For males, the top five best predictors in sequential order were age, SM, WC, VF, and AVI, however, in females, the top five predictors were age, VF, BAI, WC, and AVI. The top five for the total population were age, VF, WC, AVI, and CI.

In Table 5, the results of the ability of the various measures to discriminate between hypertension and prehypertension are presented. Generally, lower AUC values were recorded here than when the predictive value for hypertension was investigated. In males, the best five predictors in sequential order are RMR, BMI, %BF, VF, and weight, whereas the top five predictors in females are WC, AVI, weight, RMR, and CI.

## 4. Discussion

In this study, we found that about a third of the population had developed hypertension or prehypertension, respectively. It is a major public health concern. The effective control and management of hypertension are key in the steps aimed at achieving the Sustainable Development Goal 3.4, which seeks to reduce death from noncommunicable diseases by a third in 2030 relative to the 2015 figure [28]. It is important because hypertension is not only a disease state but also a significant risk factor for other noncommunicable diseases, such as cardiovascular disease, chronic kidney disease, stroke, and peripheral vascular disease [1]. Hence, research aimed at identifying the risk factors and their diagnostic/preventive potential for hypertension is needed to reduce hypertension-related mortality and morbidity.

Except for the skeletal muscle mass, the mean values of all the indices measured increased from normotensive to prehypertension, with peak values reported for hypertension. These results are similar to those reported previously in Brazilian, Chinese, and Nigerian study populations, reiterating the significant role obesity plays in the development of hypertension [15, 29, 30]. This observation, coupled with those from other studies that have reported that interventions aimed at reducing obesity lead to a significant drop in blood pressure levels, suggests that the control of obesity is one of the key targets in the fight against hypertension [31].

The waist circumference (WC), % body fat (%BF), body mass index (BMI), conicity index (CI), abdominal volume index (AVI), and body adiposity index (BAI) were higher in females than in males. Similar patterns were reported from several other population-based studies [15, 32]. These gender-based differences could stem from endocrine and genetic basis. Hence, for the same amount of fat distribution (especially for those attributable to central obesity), men have a higher cardiometabolic risk than women. Relatively lower activity levels in females than in males could be partly responsible for the observed differences. This accession is corroborated by the resting metabolic rates and skeletal muscle masses, which showed higher values in males than in females for all the BP classes.

Correlation analysis showed that all the anthropometric and BIA indices studied correlated with systolic and diastolic BP, respectively. The correlation coefficient values greater than 0.3 were observed for age and visceral fat versus SBP for all three groups. The males also had skeletal muscle mass, showing a correlation coefficient value greater than 0.3. Generally, the measures of central obesity (WC, AVI) showed higher associations with SBP than those of general obesity (BMI, %BF). This result is in agreement with those reported previously [33, 34]. In contrast, other studies have reported that central obesity and general obesity are associated with hypertension [15, 17]. These differences suggest the need for large-scale epidemiological data to be gathered across different ethnic groups to redefine the cut-off points for these anthropometric and BIA measures. It is important in determining their utility in predicting not only hypertension risk but also the risks of other noncommunicable diseases for which obesity has been shown to be a significant risk factor.

Correlation analysis for diastolic BP (DBP), however, did not follow the same pattern as that seen with SBP. Here, the skeletal muscle mass showed the highest correlation in males, whereas the visceral fat levels showed the highest correlation for females and the total study population. These differences in associations for SBP and DBP are not unexpected since the differential control of SBP and DBP has been reported in the literature [35].

To determine the predictive potential of these indices for hypertension, we conducted receiver operator analysis. From the area under the curve (AUC) values, we can see that age was the best predictor for hypertension in all the groups examined. These results are consistent with reports from previous studies, where obesity and aging have shown significant associations in Ghanaian hypertensives [36, 37]. In a Malaysia cohort, Cheah et al. showed that there was a significant association between hypertension and visceral fat [38]. Wang et al. and Goswami et al. have reported that excess visceral fat is associated with an increased risk of hypertension amongst Chinese and Indians, respectively. All these results corroborate with the results of this study [39, 40]. Our finding that the skeletal muscle mass was highly associated with and showed a better predictive value in males than females, however, contradicts that by Han et al. that showed an association between the skeletal muscle mass and hypertension that was independent of gender [41]. Because of the paucity of data on the link between hypertension and skeletal muscle mass, it is difficult to assign reasons for this observation.

Unlike hypertension, prehypertension is often asymptomatic, and although not considered a disease state in some settings, it is a significant risk factor for the development of hypertension, target-organ damage, disability from cardiovascular causes, and death [42]. Prehypertension is, therefore, a significant public health challenge. The results from this study show that prehypertension is a significant public health challenge. Studies have shown that both conditions have shared risk factors. Hence, we investigated the predictive value of all the various indices. Generally, the AUC values for all the indices were lower for prehypertension than those obtained for hypertension, which is similar to what had been previously reported [15]. None of them showed a strong predictive value for hypertension, suggesting that these indices are better predictors of hypertension than prehypertension. Resting metabolic rate, however, showed moderate predictive value for prehypertension. A recent report showed that lower educational status, sedentary lifestyle, and alcohol consumption were the predominant risk factors for prehypertension amongst study participants in Kumasi [43]. Since a direct link has been shown between a sedentary lifestyle and resting metabolic rate, these results reiterated the importance of exercise in blood pressure control [44]. However, since data on the physical activity level of study participants was not obtained in this study, the relationship between the physical activity level, resting metabolic rate, and prehypertension could not be made for this study cohort.

## 5. Conclusion

This study shows that about a third of the study participants had hypertension, with only about 33% of them having knowledge about their blood pressure status. The prevalence of prehypertension, a risk factor for hypertension, was 30.5%. Aging and visceral fat levels showed the highest association with systolic blood pressure in both genders, however, for diastolic blood pressure, these associations were gender-specific. The skeletal muscle mass showed a higher association in males, except that it was negatively correlated with SBP. Generally, the measures of central obesity showed better predictive value than those of general obesity. All the indices investigated showed a lower predictive value in prehypertension, however, RMR and weight were amongst the top five predictors of prehypertension in both genders. Future studies aimed at determining how measures to reduce central obesity can minimize the risk of hypertension are needed to determine if they may be playing a causal role. It will be important in the management and control of hypertension.

## Figures and Tables

**Table 1:** Anthropometric characteristics of study participants.

General population	Normal	Prehypertensive	Hypertensive	Total	*P*-value
Number of participants	310 (38.2%)	248 (30.5%)	254 (31.3%)	812	
Age (yrs)	40.88 ± 15.26^a^	46.77 ± 16.36^b^	55.91 ± 15.26^c^	47.39 ± 16.79	<0.0001
Weight (kg)	59.71 ± 11.82^a^	66.45 ± 16.37^bc^	67.97 ± 17.43^bc^	64.35 ± 15.60	<0.0001
Waist circumference (WC) in m	0.83 ± 0.12^a^	0.90 ± 0.15^bc^	0.93 ± 0.15^bc^	0.88 ± 0.15	<0.0001
Hip circumference (HC) in m	0. 96 ± 11.52^a^	1.00 ± 14.72^bc^	1.03 ± 15.11^bc^	0. 99 ± 14.02	<0.0001
Skeletal muscle mass (SM) in %	30.16 ± 6.55^ab^	29.26 ± 6.70^bc^	28.24 ± 6.43^cd^	29.29 ± 6.60	0.0020
Visceral fat level (VF)	5.52 ± 2.76^a^	7.50 ± 4.19^b^	8.88 ± 4.80^c^	7.18 ± 4.17	<0.0001
Resting metabolic rate (RMR)	1327.30 ± 163.43^a^	1408.38 ± 201.13^bc^	1412.23 ± 214.75^bc^	1378.51 ± 196.20	<0.0001
Body fat (BF) in %	29.54 ± 11.36^a^	32.32 ± 12.53^bc^	33.36 ± 12.68^bc^	31.59 ± 12.24	<0.0001
Body mass index (BMI) in kg/m^2^	23.45 ± 4.77^a^	25.76 ± 6.69^bc^	27.15 ± 8.41^bc^	25.31 ± 6.84	<0.0001
Conicity index (CI)	1.26 ± 0.14^a^	1.29 ± 0.15^bc^	1.32 ± 0.14^bc^	1.29 ± 0.15	<0.0001
Abdominal volume index (AVI)	14.43 ± 3.74^a^	16.84 ± 5.30^bc^	17.95 ± 5.78^bc^	16.27 ± 5.16	<0.0001
Body adiposity index (BAI)	28.89 ± 7.93^a^	30.17 ± 9.63^b^	34.45 ± 11.21^c^	31.87 ± 8.14	<0.0001

**Table 2:** Anthropometric characteristics of study participants based on gender.

	Male			Female		
	Normal	Prehypertensive	Hypertensive	Normal	Prehypertensive	Hypertensive
Number of participant	66	69	69	245	179	184
Systolic	108.99 ± 8.03	125.35 ± 9.41	152.94 ± 17.414	105.72 ± 9.16	124.94 ± 7.80	157.12 ± 21.65
Diastolic	67.68 ± 7.11	77.90 ± 8.20	91.83 ± 12.96	67.95 ± 6.63	79.87 ± 5.65	96.35 ± 12.33^*∗*^
Age	37.68 ± 16.82	45.13 ± 19.60	58.56 ± 18.79	41.70 ± 14.76	48.16 ± 14.93	54.99 ± 14.10
Weight	61.54 ± 8.43	67.09 ± 14.88	67.37 ± 14.54	59.22 ± 12.53	66.81 ± 17.59	67.66 ± 17.90
WC (m)	0.80 ± 0.07	0.86 ± 0.14	0.88 ± 0.13	0.84 ± 0.13^*∗*^	0.91 ± 0.15^*∗*^	0.95 ± 0.16^*∗*^
HC (m)	0.92 ± 6.69	0.94 ± 16.49	0.97 ± 15.18	0.97 ± 12.27^*∗*^	1.02 ± 13.24^*∗*^	1.05 ± 14.49^*∗*^
SM (%)	39.22 ± 6.76	36.72 ± 5.80	33.52 ± 6.54	27.74 ± 3.78^*∗*^	26.41 ± 4.46^*∗*^	26.23 ± 5.14^*∗*^
VF	4.91 ± 2.98	7.87 ± 4.97	8.67 ± 5.10	5.69 ± 2.68^*∗*^	7.36 ± 3.86	8.96 ± 4.69
RMR	1488.83 ± 154.08	1546.68 ± 176.47	1511.23 ± 212.92	1284.22 ± 136.96^*∗*^	1354.76 ± 184.15^*∗*^	1374.69 ± 203.75^*∗*^
BF (%)	17.45 ± 8.77	22.41 ± 9.56	23.55 ± 10.90	32.72 ± 9.72^*∗*^	36.14 ± 11.42^*∗*^	37.05 ± 11.27^*∗*^
BMI (kg/m^2^)	21.85 ± 3.01	24.25 ± 5.64	24.24 ± 5.73	23.87 ± 5.05^*∗*^	26.35 ± 6.98^*∗*^	28.26 ± 9.00^*∗*^
CI	1.23 ± 0.09	1.24 ± 0.13	1.28 ± 0.13	1.27 ± 0.15^*∗*^	1.31 ± 0.16^*∗*^	1.33 ± 0.15^*∗*^
AVI	13.24 ± 2.05	15.39 ± 5.64	16.14 ± 4.94	14.74 ± 4.02^*∗*^	17.40 ± 5.07^*∗*^	18.64 ± 5.94^*∗*^
BAI	24.64 ± 4.58	26.48 ± 7.12	27.42 ± 8.41	31.58 ± 6.00^*∗*^	33.77 ± 6.82^*∗*^	36.63 ± 9.16^*∗*^

^*∗*^ *p* < 0.05 vs. normal; ^#^*p* < 0.05 vs. prehypertensive.

**Table 3:** Correlation tables of the association between different anthropometric and BIA indices and blood pressure.

A. Total population				
	Systolic blood pressure		Diastolic blood pressure	
	*R*	*p*-value	*R*	*p*-value
Age	0.414	<0.0001	0.252	<0.0001
Weight	0.172	<0.0001	0.223	<0.0001
WC	0.271	<0.0001	0.264	<0.0001
HC	0.202	<0.0001	0.241	<0.0001
SM	−0.126	<0.0001	−0.197	<0.0001
VF	0.340	<0.0001	0.308	<0.0001
RMR	0.111	0.0020	0.124	<0.0001
BF	0.108	0.0020	0.189	<0.0001
BMI	0.209	<0.0001	0.239	<0.0001
CI	0.191	<0.0001	0.137	<0.0001
AVI	0.278	<0.0001	0.280	<0.0001
BAI	0.237	<0.0001	0.252	<0.0001

B. Male				
Age	0.4567	<0.0001	0.3201	<0.0001
Weight	0.1699	0.0151	0.1991	<0.0001
WC	0.2772	<0.0001	0.2614	0.0002
HC	0.1719	0.0142	0.1626	0.0204
SM	−0.3729	<0.0001	−0.3483	<0.0001
VF	0.3613	<0.0001	0.3182	<0.0001
RMR	0.0345	0.6248	0.0743	0.2923
BF	0.2238	0.0014	0.2836	<0.0001
BMI	0.1893	0.0067	0.1927	0.0057
CI	0.2198	0.0016	0.1973	0.0047
AVI	0.2620	0.0002	0.201	0.0002
BAI	0.1556	0.0547	0.1161	0.1528

C. Female				
Age	0.4091	<0.0001	0.228	<0.0001
Weight	0.1713	<0.0001	0.2306	<0.0001
WC	0.2813	<0.0001	0.2663	<0.0001
HC	0.2312	<0.0001	0.2686	<0.0001
SM	−0.133	0.0011	−0.1928	<0.0001
VF	0.3373	<0.0001	0.3058	<0.0001
RMR	0.1214	0.0020	0.1670	<0.0001
BF	0.1323	<0.0001	0.1849	<0.0001
BMI	0.2258	<0.0001	0.2514	<0.0001
CI	0.1940	<0.0001	0.1202	0.0030
AVI	0.2952	<0.0001	0.2858	<0.0001
BAI	0.2675	<0.0001	0.2749	<0.0001

**Table 4:** Receiver-operator characteristics results for predicting hypertension.

A. Total population					
Variable	Sensitivity	Specificity	Accuracy	AUC	*p*-value
Age	0.6482	0.6883	0.6757	0.7153	<0.0001
Weight	0.5709	0.5871	0.5820	0.5885	<0.0001
WC	0.5630	0.6661	0.6338	0.6325	<0.0001
HC	0.5099	0.6613	0.6139	0.6029	<0.0001
SM	0.2988	0.8628	0.6870	0.5756	0.0003
VF	0.3773	0.8579	0.7079	0.6572	<0.0001
RMR	0.5259	0.5935	0.5725	0.5644	0.0034
%BF	0.2698	0.8613	0.6766	0.5616	0.0049
BMI	0.3858	0.7742	0.6527	0.6009	<0.0001
CI	0.6378	0.5314	0.5647	0.6105	<0.0001
AVI	0.5630	0.6613	0.6305	0.6315	<0.0001
BAI	0.6680	0.4846	0.5422	0.6000	<0.0001

B. Male					
Age	0.8116	0.6285	0.6915	0.7500	<0.0001
Weight	0.2857	0.8806	0.6765	0.5531	0.2358
WC	0.5286	0.6767	0.6256	0.6389	0.0002
HC	0.2857	0.8797	0.6749	0.5708	0.0847
SM	0.7971	0.5263	0.6188	0.6963	<0.0001
VF	0.6232	0.6045	0.6108	0.6347	<0.0001
RMR	0.2029	0.9254	0.6798	0.4692	0.5038
%BF	0.6087	0.5564	0.5743	0.5908	0.0317
BMI	0.4714	0.6866	0.6127	0.5637	0.1396
CI	0.6571	0.5672	0.5980	0.6334	0.0004
AVI	0.5714	0.6642	0.6324	0.6345	0.0009
BAI	0.8143	0.3636	0.5198	0.5787	0.0623

C. Female					
Age	0.6413	0.6690	0.6606	0.6980	<0.0001
Weight	0.5761	0.6052	0.5964	0.5993	<0.0001
WC	0.6250	0.6250	0.6250	0.6418	<0.0001
HC	0.7322	0.4621	0.5438	0.6222	<0.0001
SM	0.3626	08290	0.6882	0.5912	<0.0001
VF	0.3934	0.8649	0.7123	0.6671	<0.0001
RMR	0.4560	0.7227	0.6424	0.5911	0.0005
%BF	0.3388	0.8223	0.6760	0.5832	0.0009
BMI	0.5163	0.6651	0.6201	0.6179	<0.0001
CI	0.7065	0.4846	0.5519	0.6150	<0.0001
AVI	0.6196	0.6274	0.6250	0.6416	<0.0001
BAI	0.8361	0.3729	0.5132	0.6430	<0.0001

**Table 5:** Receiver-operator characteristics results for predicting prehypertension.

A. Total population					
Variable	Sensitivity	Specificity	Accuracy	AUC	*p*-value
Age	0.6842	0.3316	0.4394	0.4874	0.5267
Weight	0.6154	0.5372	0.5610	0.5626	0.0050
WC	0.5968	0.5471	0.5623	0.5531	0.0075
HC	0.2984	0.7804	0.6324	0.5235	0.2575
SM	0.4878	0.5528	0.5329	0.4989	0.9571
VF	0.6694	0.4286	0.5625	0.5479	0.0307
RMR	0.6235	0.5357	0.5626	0.5710	0.0015
%BF	0.3710	0.7084	0.6647	0.5249	0.2533
BMI	0.5242	0.5833	0.5653	0.5423	0.0586
CI	0.4899	0.5957	0.5635	0.5272	0.1340
AVI	0.5887	0.5603	0.5690	0.5508	0.0230
BAI	0.4899	0.5456	0.5285	0.5010	0.9325

B. Male					
Age	0.8696	0.1591	0.4030	0.4299	0.0883
Weight	0.6522	0.5111	0.5588	0.5596	0.1601
WC	0.4348	0.7463	0.6404	0.5104	0.8154
HC	0.4928	0.6642	0.6059	0.5091	0.8374
SM	0.5882	0.5075	0.5347	0.5030	0.9425
VF	0.4638	0.6493	0.5862	0.5656	0.0057
RMR	0.6232	0.6119	0.6158	0.5837	0.0444
%BF	0.4928	0.6842	0.6188	0.5702	0.0973
BMI	0.4348	0.7481	0.6422	0.5761	0.0752
CI	0.2899	0.7704	0.6078	0.4647	0.4011
AVI	0.4638	0.7407	0.6471	0.5113	0.8085
BAI	0.4265	0.7090	0.6139	0.5290	0.5146

C. Female					
Age	0.7697	0.3124	0.4465	0.5184	0.4297
Weight	0.4270	0.7319	0.6425	0.5611	0.0220
WC	0.6425	0.4988	0.5411	0.5705	0.0027
HC	0.3855	0.7418	0.6364	0.5427	0.0841
SM	0.6461	0.4518	0.5091	0.5366	0.0683
VF	0.6816	0.4155	0.4942	0.5422	0.1038
RMR	0.5281	0.6408	0.6076	0.5594	0.0253
%BF	0.5084	0.6362	0.5983	0.5504	0.0463
BMI	0.4469	0.6690	0.6036	0.5411	0.1199
CI	0.7753	0.3403	04679	0.5534	0.0137
AVI	0.6369	0.5082	0.5461	0.5665	0.0092
BAI	0.6313	0.4376	0.4950	0.5120	0.6442

## Data Availability

All data reported are included in the manuscript. Any clarification and additional requests can be made to the corresponding author.
